# The association of the STarT Back Screening Tool and type of leg pain with low back pain disability trajectories: a prospective cohort study

**DOI:** 10.1186/s12891-024-07301-8

**Published:** 2024-03-04

**Authors:** Gijs P.G. Lemmers, René J.F. Melis, Sophie Pagen, Robin Hak, Ellen K. de Snoo, Gert P. Westert, Philip J. van der Wees, J. Bart Staal

**Affiliations:** 1 Radboud University Medical Center, IQ Health, Kapittelweg 54, Nijmegen, 6525 EP The Netherlands; 2 Dutch Healthcare Authority, Utrecht, The Netherlands; 3 Department of Geriatric Medicine, Radboud University Medical Center, Nijmegen, The Netherlands; 4 The Fysioclub, Gemert, The Netherlands; 5 Fysius Back Experts, Nijverdal, The Netherlands; 6 ProReha Fysiotherapie, Wassenaar, The Netherlands; 7 Musculoskeletal Rehabilitation Research Group, HAN University of Applied Sciences, Nijmegen, The Netherlands

**Keywords:** Low back pain, Disability, SBST, Leg pain, Physiotherapy

## Abstract

**Background:**

Multiple factors influence the recovery process of low back pain (LBP). The identification and increased knowledge of prognostic factors might contribute to a better understanding of the course of LBP. The purpose of this study is to investigate the association of the STarT Back Screening Tool (SBST) risk score and the type of leg pain (non-radiating LBP, referred non-radicular, and radicular radiating leg pain) with the disability trajectory (at baseline, the slope, and recovery at one year) in adults with low back pain.

**Methods:**

This is a prospective cohort study in 347 patients with low back pain who sought physiotherapy care at three primary care practices in the Netherlands. Linear mixed models were estimated to describe the association of the SBST risk score and the type of leg pain with disability at baseline, the slope in the disability trajectory, and at twelve months follow-up.

**Results:**

A medium/high risk score on the SBST is associated with higher baseline disability scores on the Oswestry Disability Index (ODI), faster initial recovery, and still a higher disability ODI score at 12 months follow-up. Non-radicular referred and radicular radiating leg pain were associated with worse baseline disability ODI scores in LBP. This association was not present for the initial recovery or at the 12 months follow-up.

**Conclusion:**

The SBST is associated with the LBP recovery trajectory. The SBST might be a useful tool to predict the disability trajectory in a heterogeneous group of people with low back pain in primary care and might, therefore, be recommended in future clinical practice guidelines. The type of leg pain was not associated with the recovery trajectory of LBP. Future research might focus on evaluating different types of leg pain.

**Trial registration:**

Clinicaltrials.gov: 109,643.

**Supplementary Information:**

The online version contains supplementary material available at 10.1186/s12891-024-07301-8.

## Introduction

Low back pain is costly and the disease burden is enormous worldwide [1, 2]. When encountering low back pain, self-management and physiotherapy are recommended in clinical practice guidelines [3, 4]. People with low back pain consist of a heterogeneous population with substantial variability in prognosis where psychosocial prognostic factors (e.g. stress, fear, depression, and anxiety) and physical prognostic factors (e.g. high baseline pain, high baseline disability, physically demanding labour, and sedentary behaviour) are explicitly mentioned in low back pain guidelines [5, 6]. The STarT Back Screening Tool (SBST) is a prognostic tool measuring five psychosocial items and four physical items that may support prognosis and clinical decision making [7]. Another prognostic factor is the presence of leg pain which can be of radicular or non-radicular origin [3, 4]. Remarkably, some recent guidelines no longer distinguish between low back pain with or without leg pain as there is no clear evidence whether the course of low back pain with or without leg pain is different [3, 4]. More clarity about the association of the SBST risk score and the distinction of the type of leg pain with the course of low back pain might contribute to an increase of knowledge on the course of the low back pain, adjustment of treatment, and to inform future guidelines.

The SBST risk score is used to assign patients’ risk of long-term low back pain-related disability to a low, medium, or high-risk category [7]. Several randomized controlled trials [8–10] separated people with back pain into distinct categories of risk for persistent disabling back pain. Multiple cohort studies [11–18] reported associations of SBST subgroups with a higher risk for poorer clinical outcomes. However, a recent meta-analysis reported that for patient-reported pain intensity and disability, there is insufficient evidence supporting the use of classification systems above generalized interventions when managing low back pain [19]. 

Several systematic reviews and cohort studies reported less favourable outcomes for people with low back pain including radicular complaints in the leg versus people with low back pain [20–25]. However, other systematic reviews reported no differences or an unclear association in the recovery trajectory between people with and people without radicular complaints in the leg [26–28]. Classification systems used in these studies vary a lot, and few of them focus on distinguishing different types of leg pain, showing the need for further research into type of leg pain-subgroups based on non-radiating low back pain, non-radicular referred low back pain, and radicular radiating low back pain [26, 29]. 

In summary, there is uncertainty about the long-term low back pain disability trajectory according to the SBST risk score and the type of leg pain. The purpose of this study was to investigate the association of the SBST risk score and the type of leg pain, with the disability trajectory (at baseline, the recovery slope, and recovery at one year) in adults with low back pain seeking primary care. We hypothesized that participants with a higher risk score on the SBST or radiating leg pain show a higher baseline disability score on the Oswestry Disability Index (ODI), a slower recovery, and a worse disability score on the ODI at 12 months follow-up compared to participants with a lower risk on the SBST or to participants with non-radiating low back pain.

## Methods

### Design

In this prospective cohort study, participants were enrolled by twenty physiotherapists who were employed at three primary care physiotherapy practices specialized in back and neck complaints, located in three cities in the Netherlands. The inclusion of participants occurred between June 2020 and June 2021. Follow-up data were collected at one and a half, three, six, and twelve months. The Central Committee on Research Involving Human Subjects (RadboudUMC 2020–6295) approved this study. For the reporting in this study, the STROBE guidelines were applied [30]. This study was performed in concordance with the Declaration of Helsinki.

### Participants

All patients with low back pain who were at least eighteen years old and visited through direct access or were referred by a physician or doctor for physiotherapy treatment were invited to participate. Low back pain (LBP) is defined as pain between the lower edge of the ribs and the buttocks [31]. Various types of low back pain, e.g., radiculopathy, previous surgery, were included. Individuals were not enrolled if they were unable to complete questionnaires and in case of pregnancy. Pregnancy was an exclusion criterion because it highly influences the course of low back pain. No other exclusion criteria were used. The presence of mental illness was not used as an exclusion criterion to represent a heterogeneous and representative population and we included mental aspects in the data analysis. Before enrolment, written informed consent was signed by all participants. Usual physiotherapy care based on the recommendations of the national physiotherapy guideline for low back pain was applied to all participants [4]. The number of treatment sessions and type of treatment were based on individual needs, ranging from manual therapy to exercise therapy to education whether or not in parallel. No other healthcare professionals were involved during the physiotherapy treatment. Details on healthcare utilization after physical therapy treatment are unknown. The content of the physiotherapy was not controlled for in this study to stay as close to daily practice as possible.

### Measurements

The baseline measurements for each participant included educational level, age, gender, duration of low back pain of the current episode of LBP, and the number of previous episodes of low back pain. These data were collected digitally in the electronic health record system. In addition, three questionnaires regarding pain (NPRS), disability (ODI), and psychosocial prognostic factors (SBST) were completed.

The main independent variables of interest were prognostic factors measured with the SBST and the type of leg pain. The Dutch Version of the SBST was used for an impression of the risk of developing long-term disability [32]. The SBST is a valid and reliable risk stratification tool, which categorizes people based on the total score of nine questions [7]. Questions one to four address physical factors, and questions five to nine address psychosocial factors. The risk score is categorized into a low, medium, or high risk of developing persistent disabling low back pain [33]. At the first appointment with the physiotherapist, the type of leg pain of the participant was assessed by the physiotherapist. The three pain subgroups based on non-radiating low back pain (LBP between the lower margin of the 12th rib as the upper limit and above the iliac crest and the sacral bone as the lower limit) [31], non-radicular referred leg pain (LBP lower than the upper part of the iliac crest and intervertebral disc L5-S1 but not below the knee (e.g., buttocks, thighs) and Straight Leg Raise negative), and radicular radiating leg pain (LBP with radicular complaints below the knee and Straight Leg Raise positive) were pre-defined by the researchers [34]. The researchers trained the physiotherapists who performed the physical tests in the participants with LBP to optimise reliability of testing [35, 36]. 

Perceived disability as the main outcome of interest was the dependent variable, measured with the Dutch version of the Oswestry Disability Index (ODI) [37]. The ODI has established psychometric properties and was used to assess pain-related disability in people with low back pain [37, 38]. The total score of the ODI ranged from 0 (no limitation) to 100 (bed-bound or dramatic limitation) [38, 39]. The Minimal Clinically Important Change (MCIC) has been reported to be six points or 30% improvement from baseline [38, 39]. The ODI was measured by means of a digital questionnaire at one and a half, three, six, and twelve months follow-up. In case of an incomplete measurement, the participants were contacted by their physiotherapist via telephone or email with a request to complete the questionnaires. When after 48 h the questionnaires were not completed, they were contacted by the coordinating researcher. The reason for loss to follow-up was registered by the physiotherapist.

### Data analysis

General linear mixed models were used to describe the association of the risk for long-term disability (SBST) and the type of leg pain at the start of physiotherapy, with disability (ODI) trajectories over follow-up at one and a half, three, six, and twelve months. Predictors at baseline were the type of leg pain and the SBST risk score. Gender, education level, age, pain, number of previous episodes of low back pain, and duration of low back pain were analysed as additional predictors in the regression analyses. There is some evidence that these factors could influence the course of LBP [5, 6]. Within the multilevel modelling framework, individual growth modelling was applied to the data [40, 41]. R version 5.12.10 was used for descriptive statistics and the general linear mixed models (lme4). Unconditional growth models with random effects were composed including unstructured variance-covariance matrices. The fit of the model was compared using likelihood ratio testing for nested models. Based on the observed trajectories and fit statistics for an unconditional quadratic growth model across the full 12 months follow-up, we additionally explored a spline growth model, that allowed the trajectory between 6 and 12 months to deviate from the trajectory over the first 6 months. These spline models are explicitly used to model non-linearity beyond the flexibility that can be achieved with quadratic growth models. This provides a suitable transformation for “correcting” nonlinearity in longitudinal data using the same methods used for accounting for nonlinearity in cross-sectional data. Rather than examining a single “outcome vs. predictor” plot, however, multiple empirical growth plots are examined, one for each sample member, seeking a transformation that works adequately for almost everyone under study. The fit statistics of the quadratic growth model with a spline to allow a deviation at 12 months follow up from the individually predicted outcome score at 6 months follow up exceeded the fit statistics of the model without a spline and made sense clinically. Our predictors of interest and the covariates were added to the best unconditional growth model in a second step. Differences on baseline (intercept) and the slope (linear and quadratic rate), and the difference in ODI scores at 12 months follow-up were researched. Full maximum likelihood was used for building the models and restricted maximum likelihood was used in the final model that investigated the influence of our predictors on the average growth parameters. When data on one of the predictors SBST and leg pain was missing, cases were left out of the analysis. Missing on ODI score at one of the follow-up timepoints was handled by the linear mixed models. Linear mixed effects models (LMMs) offer a simple alternative to handle missing data under a missing at random assumption without requiring imputations [42]. Therefore, available ODI data were used in the analysis.

### Secondary analysis

In a secondary analysis, the participants were divided into a group with ODI scores of 22 and below and a group of ODI scores above 22 at baseline. In previous research the average ODI score of people with low back pain-related disability was 22.08 points [39]. This analysis was performed because we expected faster initial recovery in the group with worse baseline ODI scores, because these patients also need to improve more to return to the preclinical situation. As we also expected a correlation between baseline ODI and SBST scores, this would result in higher SBST risk scores being related to better rather than worse recovery rates in the whole group. The number of participants with high-risk SBST scores and radicular radiating type of leg pain were low. To improve the statistical power of this analysis the three risk score groups low, medium, and high risk of the SBST were divided into two groups of low risk and medium/high risk. The type of leg pain groups were divided into non-radiating low back pain and non-radicular referred/radicular radiating leg pain. A general linear hypotheses test (glht) was applied to check the significance of the difference between low and medium/high risk SBST groups, and the difference between non radiating low back pain and non-radicular referred/radicular radiating leg pain at 12 months follow-up.

## Results

### The flow of participants through the study

**Fig. 1:**
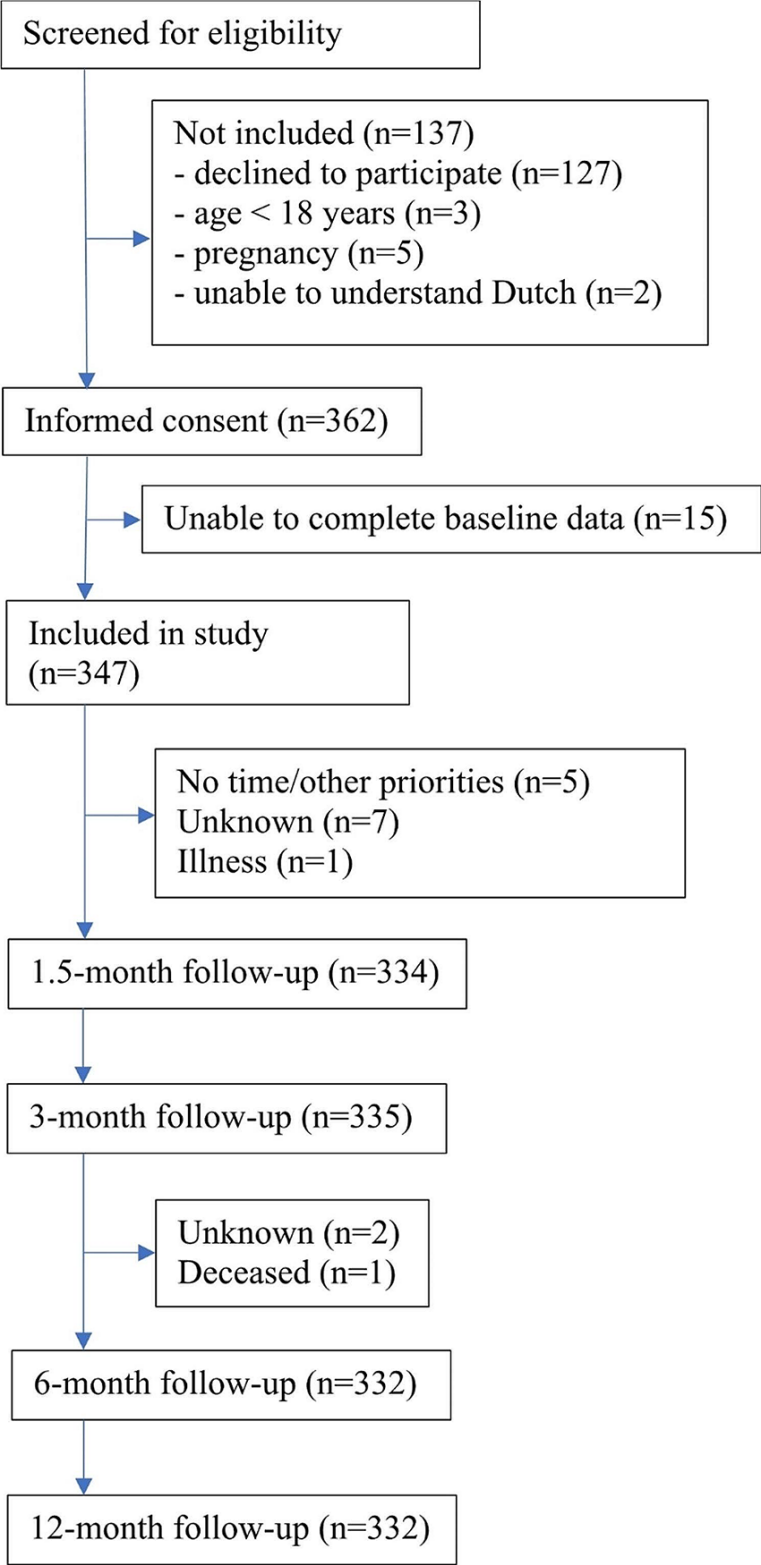
Flowchart of participants

Eligibility screening was performed for 484 consecutive potential participants. Of the potential participants, 362 were eligible and willing to participate (Fig. 1). Baseline data were not completed by 15 participants. A total of 347 participants completed the baseline data and were enrolled in the study. Until the 12-month follow-up, 332 participants (96%) completed the questionnaires (15 participants lost to follow-up).

### Characteristics of participants

The participants had a mean age of 43(SD 14.6) years (Table 1). The 347 participants consisted of 50% men and 50% women. The participants showed large differences in back pain duration at baseline. Of the participants, 275 people (79%) already had chronic low back pain defined as twelve weeks or more at baseline. The ODI had a median (IQR) outcome of 20 (10–32) at baseline. 48% of the participants had a low-risk SBST score for long-term disability, 41% had a medium risk for long-term disability, and 10% had a high-risk for long-term disability. Concerning type of leg pain, 40% had non-radiating LBP, 54% had non-radicular referred leg pain, and 6% had radicular radiating leg pain. There was substantial heterogeneity amongst the participants on the majority of the baseline characteristics, resulting in large standard deviations. Table 1 summarizes the characteristics of the participants.

**Table 1:** Characteristics of Participants (*n* = 347)

**Gender (n)**		**Questionnaires**	
Female (%)	172 (50)	ODI baseline (median)(IQR)	20 (10–32)
Male (%)	175 (50)	ODI 1.5 months (median)(IQR)	8 (2–20)
**Age in years (mean)(SD)**	43 (15)	ODI 3 months median)(IQR)	6 (0–14)
**Back pain duration at baseline (n) (%)**		ODI 6 months (median)(IQR)	4 (0–14)
0–2 weeks	25 (7)	ODI 12 months (median)(IQR)	4 (0–10)
3–12 weeks	47 (14)	NPRS baseline (mean)(SD)	5.3 (2.3)
3–6 months	21 (6)	**Outcome STarT Back Screening Tool (n) (%)**	
7–12 months	30 (9)	Low risk	166 (48)
1–4 years	91 (26)	Medium risk	143 (41)
5–9 years	60 (17)	High risk	34 (10)
10–20 years	43 (12)	**Type of leg pain (n) (%)**	
> 20 years	29 (8)	Non-radiating low back pain	137 (40)
		Non-radicular referred leg pain	186 (54)
		Radicular radiating leg pain	22 (6)

n = number, SD = Standard Deviation, ODI = Oswestry Disability Index (range 0-100), IQR = Inter Quartile Range. NPRS = Numeric Pain Rating Scale (range 0–10)

### Growth models

The best fitting unconditional growth model for the Oswestry Disability Index (ODI) over the follow-up time points included a linear and a quadratic fixed slope across the first 6 months of follow up and a spline to model the difference in ODI scores between 6 and 12 month follow up. As such, the average patient presented with a ODI score of 22 [95% CI 20 to 23] at baseline and followed a disability trajectory that initially improved with − 6.3 points [95% CI -7.1 to -5.6] on average on the ODI per month in the first six months. However, this initial improvement gradually slowed down over time with 0.7 ODI point on average per month [2]. For the average patient this resulted in a ODI score of 9 points at six months, that further decreased 1.5 ODI points from six to twelve months (model 1, Table 2). To allow individual participants to deviate from the population mean trajectory a random intercept and random linear slope were added as the differences in baseline and slopes in ODI were considerable (model 1). Negatively correlated (-0.72) random effects were found, meaning that participants with higher baseline ODI scores had higher negative linear slopes over the follow-up time points (i.e., faster recovery in ODI disability scores) (model 1).

**Table 2:** The Results on baseline and slope for Fitting Different Individual Growth Models in Disability Trajectory Outcome Oswestry Disability Index within 1-year follow-up

	Model 1: Unconditional growth model (*n* = 347)			Model 2: Effects of Type of leg pain^a^ and SBST^b^ outcome UNadjusted (*n* = 347)			Model 3: Effects of Type of leg pain and SBST outcome adjusted^c^ (*n* = 347)		
**Characteristic**	**Beta** ^**g**^	**95% CI** ^**d**^	**p-value**	**Beta** ^**g**^	**95% CI** ^**d**^	**p-value**	**Beta** ^**g**^	**95% CI** ^**d**^	**p-value**
ODI at BL	22	20, 23	< 0.001	12	9.9, 15	< 0.001	12	9.4, 15	< 0.001
SBST category									
Low				—	—		—	—	
Medium^e^				12	8.9, 15	< 0.001	11	7.8, 14	< 0.001
High				23	18, 27	< 0.001	22	17, 27	< 0.001
Type leg pain									
LBP				—	—		—	—	
Referred^f^				2.7	-0.13, 5.6	0.061	2.5	-0.64, 5.7	0.12
Radicular				12	6.4, 18	< 0.001	15	9.0, 22	< 0.001
linear change during first 6 months	-6.3	-7.1, -5.6	< 0.001	-3.6	-5.0, -2.2	< 0.001	-2.2	-3.7, -0.67	0.005
SBST category * linear change during first 6 months									
Medium * linear change during first 6 months				-5.0	-6.6, -3.3	< 0.001	-6.2	-8.0, -4.4	< 0.001
High * linear change during first 6 months				-7.0	-9.7, -4.3	< 0.001	-9.2	-12, -6.3	< 0.001
Type leg pain * linear change during first 6 months									
Referred * linear change during first 6 months				-0.05	-1.7, 1.6	> 0.9	-0.34	-2.1, 1.4	0.7
Radicular * linear change during first 6 months				0.03	-3.3, 3.4	> 0.9	-1.3	-4.9, 2.3	0.5
quadratic change during first 6 months	0.72	0.60, 0.83	< 0.001	0.41	0.20, 0.62	< 0.001	0.23	0.00, 0.45	0.053
SBST category * quadratic change during first 6 months									
Medium * quadratic change during first 6 months				0.64	0.39, 0.89	< 0.001	0.80	0.52, 1.1	< 0.001
High * quadratic change during first 6 months				0.75	0.34, 1.2	< 0.001	1.1	0.62, 1.5	< 0.001
Type leg pain * quadratic change during first 6 months									
Referred * quadratic change during first 6 months				-0.02	-0.27, 0.23	0.9	0.01	-0.26, 0.28	> 0.9
Radicular * quadratic change during first 6 months				-0.16	-0.67, 0.35	0.5	-0.04	-0.59, 0.51	0.9
difference between ODI at 6 and 12 months	-1.5	-2.9, -0.22	0.023	-1.9	-4.2, 0.44	0.11	-2.5	-5.1, 0.08	0.058
SBST category * difference between ODI at 6 and 12 months									
Medium * difference between ODI at 6 and 12 months				0.38	-2.5, 3.2	0.8	1.1	-2.1, 4.3	0.5
High * difference between ODI at 6 and 12 months				3.8	-0.93, 8.5	0.12	5.7	0.64, 11	0.027
Type leg pain * difference between ODI at 6 and 12 months									
Referred * difference between ODI at 6 and 12 months				-0.18	-3.0, 2.6	0.9	0.03	-3.0, 3.1	> 0.9
Radicular * difference between ODI at 6 and 12 months				-1.5	-7.6, 4.7	0.6	-1.9	-8.6, 4.7	0.6
random intercept (sd)	12			9.5			9.4		
correlation random effects	-0.72			-0.64			-0.67		
random linear slope (sd)	1.5			1.4			1.4		
residuals (sd)	8.7			8.6			8.4		
AIC	12,82			12,47			10,39		
BIC	12,86			12,60			10,64		

ODI = Oswestry Disability Index (dependent variable); SBST = STarT Back Screening Tool; LBP = non-radicular low back pain; AIC = Akaike information criterion; BIC = Bayesian information criterion

^a^ Non-radiating low back pain (reference category), non-radicular referred leg pain, or radicular radiating leg pain (independent variable)

^b^ STarT Back Screening Tool, Low risk (reference category), Medium risk, or High risk (independent variable)

^c^ Adjusted for baseline pain, back pain duration, gender, age, number of low back pain episodes, and education level (all independent variables)

^d^ 95% Confidence Interval

^e^ e.g., value of 12 indicates participants with a Medium risk on the SBST score 12 points higher on the ODI compared to the Low risk-participants

^f^ e.g., value of 2.7 indicates participants with non-radicular referred leg pain score 2.7 points higher on the ODI compared to the non-radiating low back pain participants

^g^ (difference on) ODI score

### Baseline (time-invariant) predictors of differences in baseline and slope in ODI scores

After fitting the unconditional growth model, we added the baseline score on the STarT Back Screening Tool and the baseline type of leg pain to test the hypotheses. We added the baseline score on the STarT Back Screening Tool and the baseline type of leg pain simultaneously as predictors of heterogeneity in the growth parameters in ODI (model 2). We added the covariables gender, education level, risk for long-term disability, age, pain, and disability, number of previous episodes of low back pain, and duration of low back pain to build an adjusted model which is the final model (model 3). Specifically, longer low back pain duration showed a statistically significant association (*p* = 0.02) with a worse linear recovery slope of low back pain in model three in this study as can be seen in supplementary Fig. 6. A statistically significant association was not present at baseline, nor in the quadratic slope, or at the 12 months follow-up. A a complete overview of all parameter estimates of model 3 are presented in supplement A.

#### Differences in STarT Back Screening Tool categories in baseline and slope in ODI scores

**Fig. 2:**
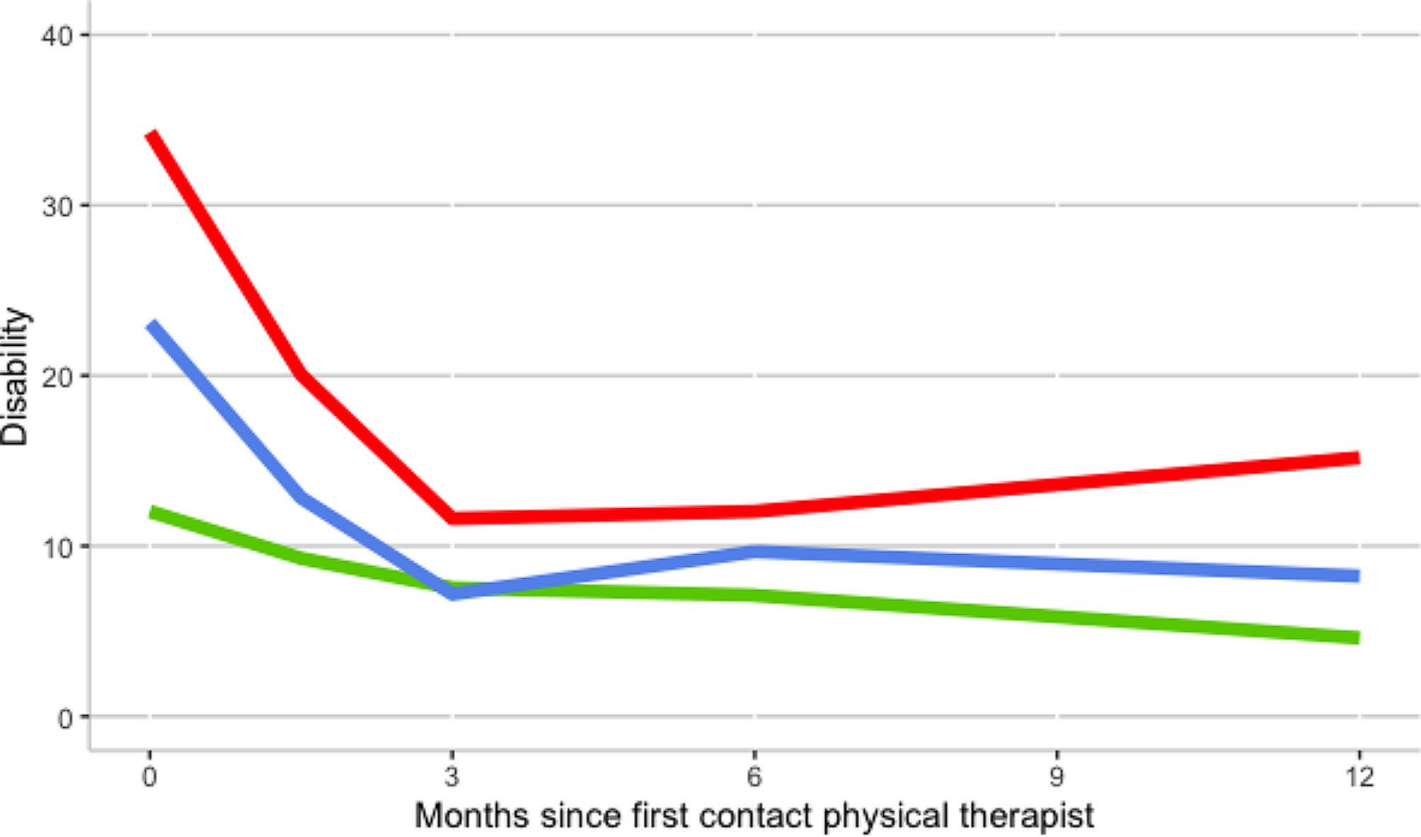
Disability trajectory of SBST categories for non-radiating LBP (model 3). Disability measured with the Oswestry Disability Index (higher score indicates worse functioning). The red line indicates the SBST high-risk group, the blue line indicates the SBST medium-risk group, the green line indicates the SBST low-risk group

At the baseline, significant differences between low, medium, and high-risk groups of the SBST were present in models two and three (Table 2). The baseline SBST risk score showed a statistically significant association with the linear and quadratic rate of change on the ODI (models 2 and 3). In model 3, average participants with non-radiating low back pain and with a low-risk score on the SBST initially recovered 2.2 [95% CI 0.67 to 3.7] points on the ODI (minus the quadratic effect) on average per month. The participants with a medium-risk score on the SBST initially recovered with 6.2 [95% CI 4.4 to 8.0] points on average per month faster on the ODI (minus the quadratic effect) compared to the low risk-participants. Participants with a high-risk score on the SBST initially recovered 9.2 [95% CI 6.3 to 12.0] points faster on the ODI on average per month compared to the participants with a low-risk score on the SBST. The quadratic rate of change for the SBST was statistically significant in models 2 and 3. This suggests that participants with a medium or high-risk score on the SBST show more slowing down of the improvement through time in comparison with participants with a low-risk score in the SBST. The patients with low-risk SBST scores at baseline further improved from 6 to 12 months FU (-2.5 [95% CI -5.1 to 0.08]), although this effect was not significantly different from zero. Particularly patients with high-risk SBST scores did no longer improve from 6 to 12 months and some even worsened again: the change from six to twelve months follow-up show a difference of 5.7 [95% CI 0.64 to 11] ODI points between the high-risk and the low-risk group (Table 2, model 3, Fig. 2).

#### Differences in STarT back screening tool categories at 12 months follow-up in ODI scores

At 12 months follow-up, participants with a high-risk baseline score on the SBST have a higher disability level with 10.6 [95% CI 5.9 to 15.2] points higher on the ODI in comparison with the participants with a low-risk score on the SBST (Table 3, contrasts as derived from model 3). Participants with a medium-risk SBST score have a 3.6 [95% CI 0.6 to 6.6] points worse 12-month ODI than the low-risk group. At 12 months follow-up, participants with a high-risk baseline score on the SBST have a higher disability level with 7.0 [95% CI 2.3 to 11.6] points higher on the ODI in comparison with the participants with a medium risk on the SBST.

#### Differences in type of leg pain in baseline, slope, and at 12 months follow-up in ODI scores

**Fig. 3:**
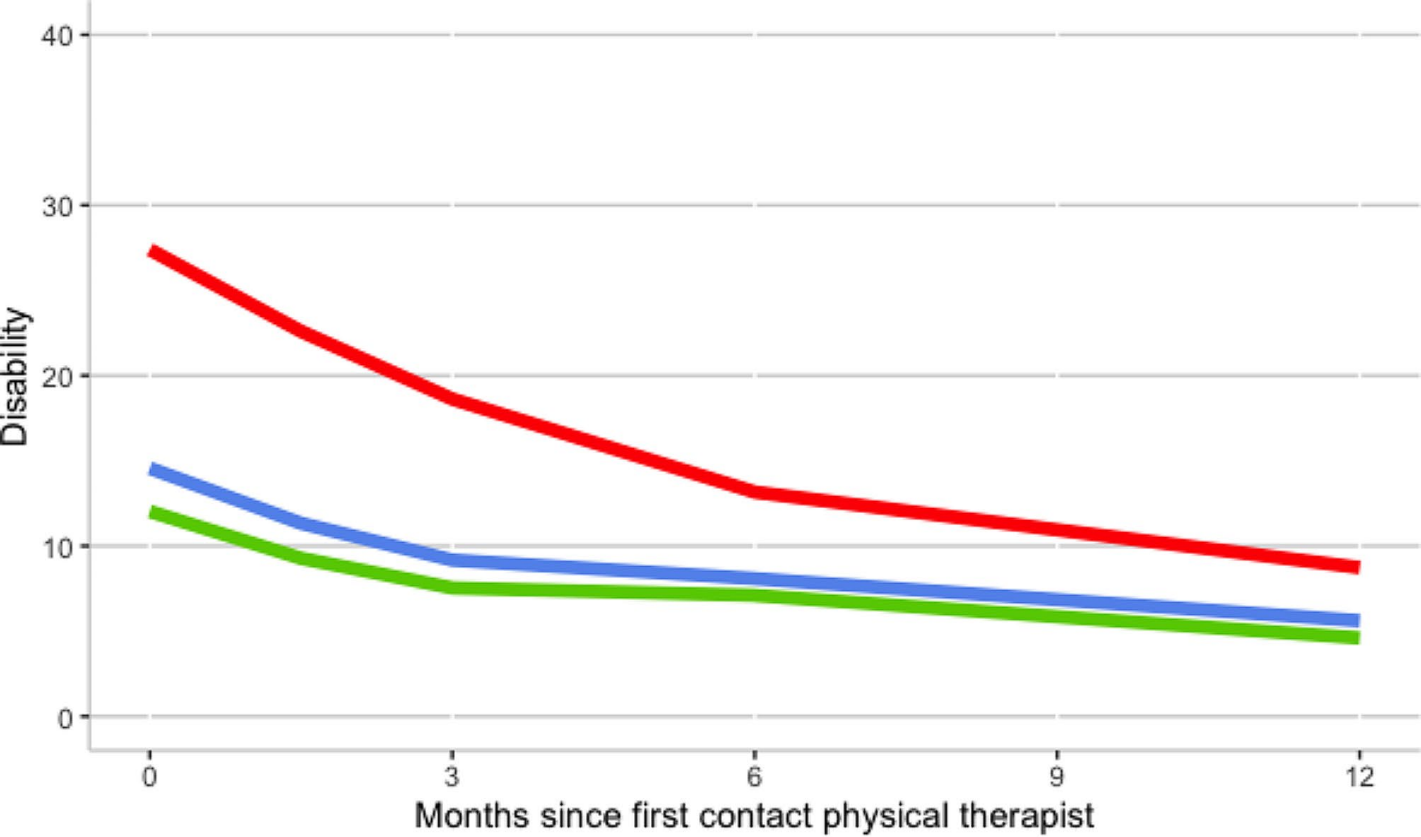
Disability trajectory of three types leg pain for low risk SBST category (model 3). Disability measured with the Oswestry Disability Index (higher score indicates worse functioning). The red line indicates the group with radicular radiating low back pain, the blue line indicates the group with radiating, non-radicular low back pain, the green line indicates the group with non-radiating low back pain

In the adjusted model (model 3) participants with radicular radiating leg pain show a 15 [95% CI 9.0 to 22.0] points higher score on the baseline ODI compared to the participants with non-radiating low back pain. Concerning the type of leg pain categories, there were no significant associations for the initial slope, the slope from 6 to 12 months follow-up, nor for the difference at 12 months follow-up between the type of leg pain-groups (Table 2 model 3, Table 3 model 3, Fig. 3).

There was no collinearity between the variables SBST and type of leg pain as the Pearson correlation was − 0.316. Figures two and three show the disability trajectories of the three categories of STarT Back Screening Tool risk score and the three types of leg pain.

**Table 3:** The Scores and Differences at 12 months follow-up in the Disability Trajectory Outcome Oswestry Disability Index

	Model 3: Effects of Type of leg pain^a^ and SBST^b^ outcome adjusted^c^ (*n* = 347)				Model 4: Secondary Analysis with high and low ODI strata (*n* = 347)		
**Characteristic**	**Beta** ^**g**^	**95% CI** ^**d**^	**p-value**	**LOW ODI STRATA** ^**e**^	**Beta** ^**g**^	**95% CI** ^**d**^	**p-value**
SBST Low^f^	4.6			SBST Low	4.22		
SBST Medium	8.2			SBST Medium/High	5.45		
SBST High	15.2			SBST Low vs. SBST Medium/High	1.22	-4.2, 6.6	0.659
SBST Low vs. SBST Medium	3.6	0.6, 6.6	0.017	Type LBP	4.23		
SBST Low vs. SBST High	10.6	5.9, 15.2	< 0.001	Type Referred/Radicular	5.82		
SBST Medium vs. SBST High	7.0	2.3, 11.6	0.003	Type LBP vs. Referred/Radicular	1.59	-3.0, 6.2	0.494
Type of leg pain LBP	4.6			**HIGH ODI STRATA** ^**e**^			
Type of leg pain Referred	5.6			SBST Low	5.44		
Type of leg pain Radicular	8.7			SBST Medium/High	10.49		
Type LBP vs. Referred	1.0	-1.8, 3.9	0.488	SBST Low vs. SBST Medium/High	5.05	1.24, 8.85	< 0.001
Type LBP vs. Radicular	4.1	-1.9, 10.2	0.182	Type LBP	5.44		
Type Referred vs. Radicular	3.1	-2.8, 9.0	0.301	Type Referred/Radicular	6.45		
				Type LBP vs. Referred/Radicular	1.01	-2.6, 4.6	0.579

^a^ Non-radiating low back pain (reference category), non-radicular referred leg pain, or radicular radiating leg pain (independent variable)

^b^ STarT Back Screening Tool, Low risk (reference category), Medium risk, or High risk (independent variable)

^c^ Adjusted for baseline pain, back pain duration, gender, age, number of low back pain episodes, and education level (all independent variables)

^d^ 95% Confidence Interval

^e^ Low: ODI ≤ 22, High: ODI > 22, differences at 12 months follow-up (dependent variable)

^f^ e.g., value of 4.6 indicates participants’ score on the ODI at 12 months follow-up

^g^ (difference on) ODI score

### The association of the SBST and type of leg pain with ODI trajectories stratified for groups of high and low baseline ODI scores

**Fig. 4:**
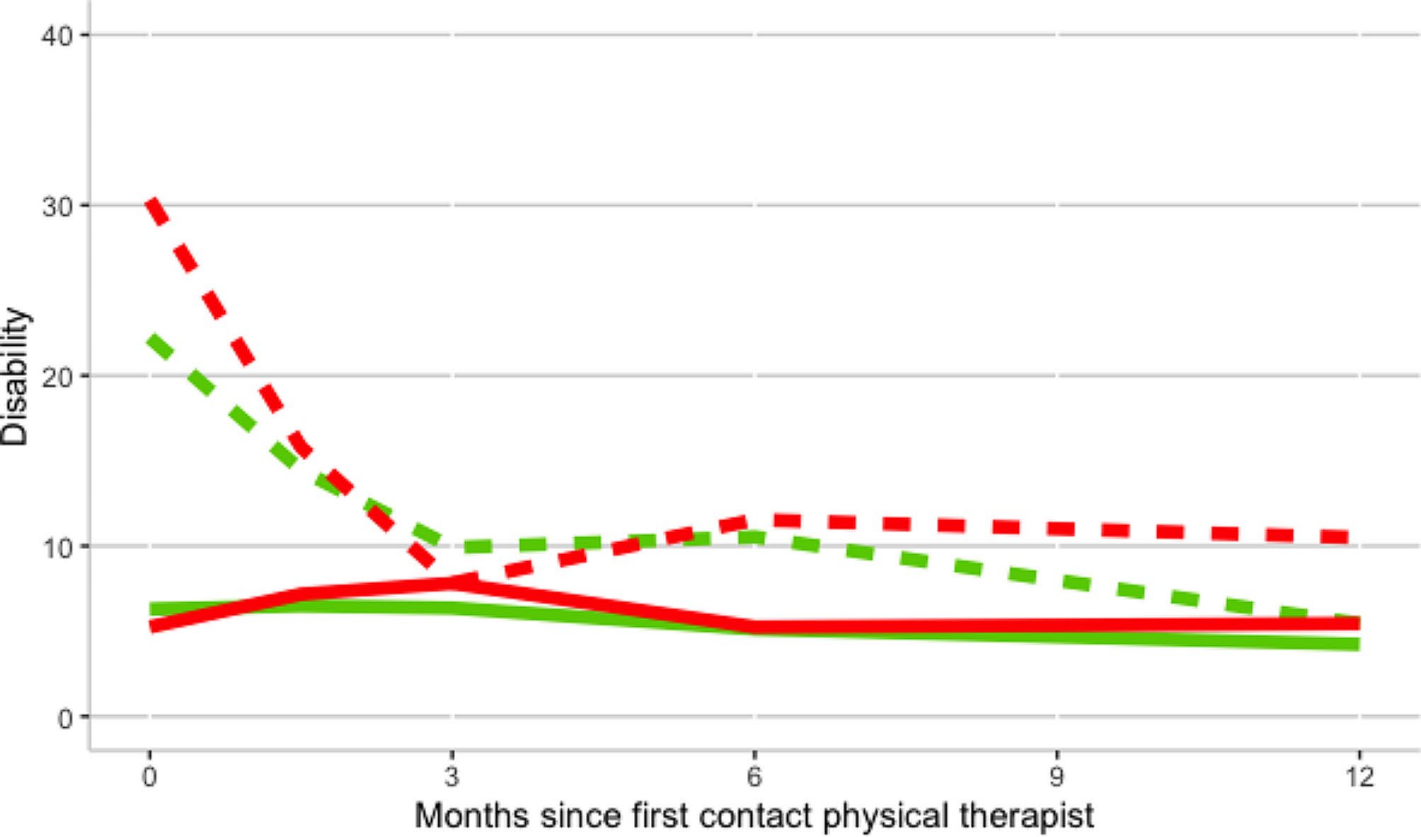
Disability trajectory of two categories of SBST for low and high ODI scores. Disability measured with the Oswestry Disability Index (higher score indicates worse functioning). The red lines represent the group with a medium/high risk score on the SBST (*n* = 177), the green lines represent the group with a low risk sore on SBST (*n* = 166). The dashed lines represent the group with high ODI scores (> 22), the continuous lines represent the group with low ODI scores (≤ 22)

**Fig. 5:**
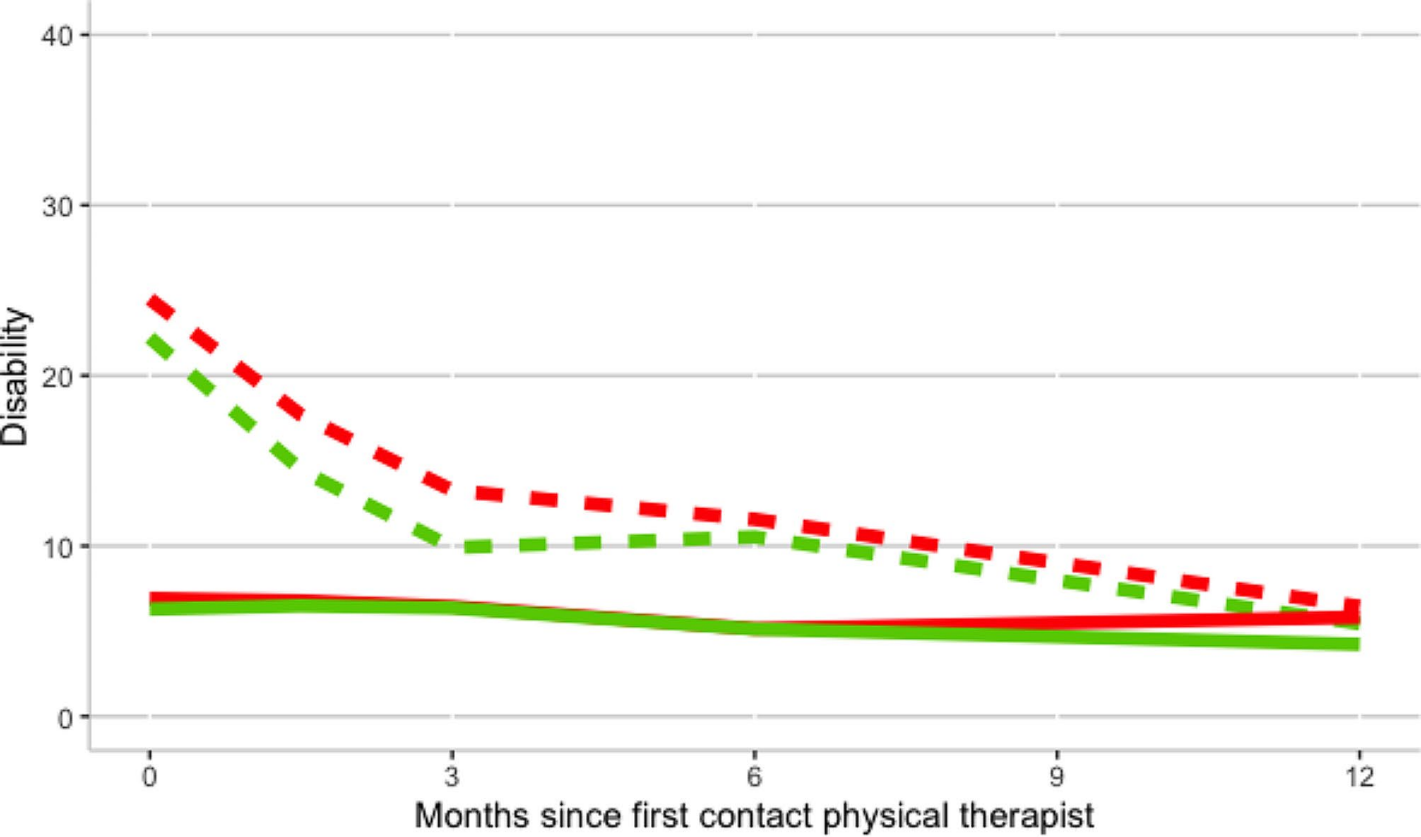
Disability trajectory of two leg pain categories for low and high ODI scores. Disability measured with the Oswestry Disability Index (higher score indicates worse functioning). The red lines represent the group with radiating (radicular and non-radicular, *n* = 208) leg pain, the green lines represent the group with non-radiating low back pain (*n* = 137) The dashed lines represent the group with high ODI scores (> 22), the continuous lines represent the group with low ODI scores (≤ 22)

After dividing the participants into groups for low (≤ 22) and high (> 22) baseline ODI scores we analysed the disability trajectories for groups with low and medium/high risk scores on the SBST (Table 4; Fig. 4), and for groups of low back pain and referred/radicular leg pain (Table 4; Fig. 5). We did so to evaluate whether SBST and type of pain could differentiate between better and worse ODI outcome (trajectories) for patients with at baseline comparable pain-related disability severity as rated using ODI.

#### Association of the STarT Back Screening Tool with ODI trajectories

The group with a medium/high risk score on the SBST within the high baseline ODI stratum showed a 9.1 [95% CI 2.7 to 16] points higher score on the ODI at baseline, a steeper decline in the first 6 months of -7.1 [95% CI -11 to -3.2] ODI points at 12 months follow-up compared to the low risk score group (Table 4). This group also had a significantly higher ODI score at 12 months follow- up of 5.05 [95% CI 1.24 to 8.85] (Table 3). The group with a medium/high risk score on the SBST within the low ODI strata shows a similar baseline score, a non-significant increase in ODI score in the first 3 months (1.5 [-1.8, 4.7], *P* = 0.4, Fig. 4), and a non-significant higher ODI score of 1.22 [95% CI -4.2 to 6.6] at 12 months follow-up compared to the low-risk score group (Tables 3 and 4).

#### Association of the type of leg pain with ODI trajectories

Differences between type of leg pain groups non-radiating LBP and non-radicular referred/radicular radiating leg pain were not statistically significant. The group with non-radicular referred/radicular radiating leg pain within the high ODI strata shows a higher baseline ODI score, lesser decline in the first 3 months (Fig. 5), and a higher ODI score of 1.01 [95% CI -2.6 to 4.6] (Table 3) at 12 months follow-up compared to the non-radiating LBP group (Fig. 5). The group with non-radicular referred/radicular radiating leg pain within the low ODI stratum shows a similar baseline ODI score, lesser decline in the first 3 months (Fig. 5), and a non-significant higher ODI score of 1.59 [95% CI -3.0 to 6.2] (Table 3) at 12 months follow-up compared to the non-radiating LBP group.

**Table 4:** Analysis of high and low ODI^a^ strata (*n* = 347)

Characteristic	Beta^d^	95% CI^c^	p-value
ODI at BL^b^	6.3	3.1, 9.5	< 0.001
ODI at BL in two groups: Low ( = < 12) & High (> 12)			
Low	—	—	
High	16	11, 21	< 0.001
SBT category: Low vs. Medium/High (independent variable)			
Low	—	—	
Medium/High	-1.0	-6.4, 4.3	0.7
Type back pain: LBP vs. Referred/Radicular (independent variable)			
LBP	—	—	
Referred/Radicular	0.62	-3.9, 5.1	0.8
Linear change during first 6 months	0.24	-1.7, 2.2	0.8
ODI at BL in two groups: Low ( = < 12) & High (> 12) * Linear change during first 6 months			
High * Linear change during first 6 months	-6.5	-9.4, -3.6	< 0.001
SBT category: Low vs. Medium/High * Linear change during first 6 months			
Medium/High * Linear change during first 6 months	1.5	-1.8, 4.7	0.4
Type back pain: LBP vs. Referred/Radicular * Linear change during first 6 months			
Referred/Radicular * Linear change during first 6 months	-0.26	-3.0, 2.5	0.9
SBT category: Low vs. Medium/High * ODI at BL in two groups: Low ( = < 12) & High (> 12) * Linear change during first 6 months			
Medium/High * High * Linear change during first 6 months	-7.1	-11, -3.2	< 0.001
ODI at BL in two groups: Low ( = < 12) & High (> 12) * Type back pain: LBP vs. Referred/Radicular * Linear change during first 6 months			
High * Referred/Radicular * Linear change during first 6 months	1.2	-2.3, 4.6	0.5
Quadratic change during first 6 months	-0.07	-0.37, 0.22	0.6
ODI at BL in two groups: Low ( = < 12) & High (> 12) * Quadratic change during first 6 months			
High * Quadratic change during first 6 months	0.79	0.36, 1.2	< 0.001
SBT category: Low vs. Medium/High * Quadratic change during first 6 months			
Medium/High * Quadratic change during first 6 months	-0.21	-0.71, 0.28	0.4
Type back pain: LBP vs. Referred/Radicular * Quadratic change during first 6 months			
Referred/Radicular * Quadratic change during first 6 months	0.03	-0.39, 0.44	> 0.9
SBT category: Low vs. Medium/High * ODI at BL in two groups: Low ( = < 12) & High (> 12) * Quadratic change during first 6 months			
Medium/High * High * Quadratic change during first 6 months	0.95	0.35, 1.5	0.002
ODI at BL in two groups: Low ( = < 12) & High (> 12) * Type back pain: LBP vs. Referred/Radicular * Quadratic change during first 6 months			
High * Referred/Radicular * Quadratic change during first 6 months	-0.22	-0.74, 0.31	0.4
Difference between ODI at 6 and 12 months	-0.89	-4.3, 2.5	0.6
ODI at BL in two groups: Low ( = < 12) & High (> 12) * Difference between ODI at 6 and 12 months			
High * Difference between ODI at 6 and 12 months	-4.2	-9.1, 0.77	0.10
SBT category: Low vs. Medium/High * Difference between ODI at 6 and 12 months			
Medium/High * Difference between ODI at 6 and 12 months	1.1	-4.6, 6.8	0.7
Type back pain: LBP vs. Referred/Radicular * Difference between ODI at 6 and 12 months			
Referred/Radicular * Difference between ODI at 6 and 12 months	1.6	-3.2, 6.3	0.5
SBT category: Low vs. Medium/High * ODI at BL in two groups: Low ( = < 12) & High (> 12) * Difference between ODI at 6 and 12 months			
Medium/High * High * Difference between ODI at 6 and 12 months	2.9	-3.8, 9.7	0.4
ODI at BL in two groups: Low ( = < 12) & High (> 12) * Type back pain: LBP vs. Referred/Radicular * Difference between ODI at 6 and 12 months			
High * Referred/Radicular * Difference between ODI at 6 and 12 months	-1.6	-7.6, 4.4	0.6
SBT category: Low vs. Medium/High * ODI at BL in two groups: Low ( = < 12) & High (> 12)			
Medium/High * High	9.1	2.7, 16	0.006
ODI at BL in two groups: Low ( = < 12) & High (> 12) * Type back pain: LBP vs. Referred/Radicular			
High * Referred/Radicular	1.6	-4.0, 7.3	0.6
random intercept (sd)	7.8		
correlation random effects	-0.50		
random linear slope (sd)	1.2		
residuals (sd)	8.1		
AIC	10,189		
BIC	10,46		

^a^ Oswestry Disability Index (dependent variable)

^b^ Baseline

^c^ Confidence Interval

^d^ (difference on) ODI score

## Discussion

### Main findings and related literature

The results of this study showed that a medium or high-risk score on the STarT Back Screening Tool (SBST) was associated with a higher baseline disability score on the ODI, faster initial recovery, and still a higher disability ODI score at 12 months follow-up, compared to a low-risk score on the SBST. The outcomes at baseline and 12 months follow-up supported our hypothesis. The change in disability in the first 3 months of the high risk-group showed a steeper improvement than the medium and low risk group. This outcome conflicted with our hypothesis. The change in disability from 6 to 12 months was significantly worse in the high-risk compared to the low-risk group, which supported our hypothesis. Radicular radiating leg pain was associated with a higher baseline score on the ODI and there is a trend for a steeper decline in the disability trajectory. These associations were not present for non-radicular referred leg pain and non-radiating low back pain. No associations of type of leg pain with ODI scores at 12 months follow-up were present. This partially supported our hypothesis of participants with radiating leg pain (referred and radicular) showing a higher baseline disability score on the ODI, showing a slower recovery, and a worse disability score on the ODI at 12 months follow-up compared to the participants with non-radiating low back pain without referred or radicular leg pain.

The result showing that the high risk SBST group showed a better initial recovery in the first three months compared to the medium and low SBST risk groups is conflicting with other cohort studies. In the study of Ben Ami et al. the the medium risk group showed a slight steeper improvement on disability outcome compared to the high risk group [11]. Furthermore, in the study of Szita et al. the high risk group showed even worse outcomes on the ODI after three months [17]. On the other hand, the high risk group of the study of Unsgaard-Tøndel et al. also showed more improvement in the first three months compared to the low risk group [18]. People with higher baseline pain or disability scores need to recover more to attain the pain or disability levels of before this LBP episode [43]. 

In the secondary analyses of high and low ODI strata we concluded that a part of the association of the SBST risk score with the LBP trajectory can be explained by the fact that participants with a high-risk score on the SBST mostly reported a high ODI disability score and thus also had more room to recover. However, within the high ODI stratum (i.e., within a group of patients that is more homogeneous in terms of ODI scores at baseline) still the medium/high risk SBST group showed a steeper disability decline in the first 6 months. Yet, the high ODI stratum also showed a higher score at 12 months follow-up compared to the low-risk group. Type of recruitment and duration of back differed between this study and previous research [44]. The study of Schuller et al. (2021) reported an average ODI score of 23.4 in a Dutch primary care setting [45]. Other studies often researched populations with less chronic low back pain, where pain and disability scores tend to be higher for patient with acute low back pain.

The course of low back pain is a common topic in scientific research, still, a lot remains unknown. We found substantial heterogeneity in the baseline values of back pain duration, age, and the disability trajectory of the Oswestry Disability Index. In this study, the mean disability trajectory for the low back pain patients showed an improvement that slowed down over the follow-up time. Stress, fear, depression, anxiety, sleep hygiene, and hard labour are factors that seem to influence the disability trajectory in low back pain [4, 5, 46]. There remains uncertainty about which factors contribute how much to what types of low back pain.

Multiple studies showed similar results on the general course of low back pain, which means a fast initial recovery which slowed down in time [44, 47, 48]. Regarding the associations of the SBST with the disability of patients with LBP, multiple studies reported similar results as in our study [11, 17, 18]. Contrary to our findings, the systematic reviews of Tagliaferri et al. [19] and Karran et al. [49] reported a lack of evidence supporting the classification systems as the SBST for the management of low back pain. However, only four trials about the SBST were included, which could have limited their results. Our study had a small group at high risk compared to medium and low risk, which is similar to other studies [10, 50, 51]. The association between the score of the SBST and the disability trajectory found in our study seems clinically relevant as the minimal clinical detectable change of the ODI of > 6 points is present for low vs. high and for medium vs. high risk groups at 12 months follow-up [52]. At twelve months follow-up, the low (4.6) and medium-risk (8.2) groups were below the functional limitations cut-off value of 12 points on the ODI, whereas the high-risk (15.2) group was above this cut-off value [39]. This implies that the people in the high-risk group, on average, still had functional limitations at twelve months follow-up and might be considered as not recovered.

No associations of type leg of pain and ODI disability scores at 12 months follow-up were present. This is in concordance with the systematic reviews of Vroomen et al., Chou et al., and Verwoerd et al. [27, 28, 53] In a cohort study, Spijker-Huiges et al. reported that the association of radicular complaints in the leg is unclear for the recovery trajectory in low back pain [26]. 

In contrast, the systematic reviews of Shaw et al. and Konstantinou et al. reported less favourable risk scores for people with low back pain including radicular complaints in the leg versus people with low back pain after a similar follow-up period [23, 25]. Shaw et al. showed that radicular pain was one of many factors to delay recovery in low back pain disability without explaining the exact size of the effect [25]. A systematic review [23] and two cohort studies [22, 24] showed that LBP patients with leg pain scored higher for measures of pain and disability at baseline and at follow-up in comparison with patients with LBP without leg pain. Perhaps in our study, the relatively small sample of people (22 participants, 6.3%) with radicular radiating leg pain played a role in the absence of an association for slopes and at 12 months follow-up score for type of leg pain, although this percentage of 6% is already somewhat high for a primary care practice.

### Strengths and limitations

This is the first quantitative study that describes associations of the low back pain disability trajectory with the score of the STarT Back Screening Tool (SBST) and with the well-defined type of leg pain. Other cohort studies investigated only either the predictive ability of the SBST or the type of leg pain in people with low back pain, never in one study [8–18, 20–25]. To investigate possible confounding effects between the type of leg pain and the SBST score it might be useful to analyse both in parallel. An important strength of our study is the growth modelling over multiple follow-up measurements with a very high follow-up percentage of 96% up to twelve months. The heterogeneous patient population is a strength considering generalizability. Internal validity is strengthened by the potential confounders that were assessed and adjusted for. However, psychosocial factors like anxiety and catastrophizing were not separately assessed, although these constructs were to some extent covered by single items of the SBST. This may have had consequences for the data analyses and the results as these factors are supposed to impede recovery in low back pain [4, 5, 54]. The researchers that performed the data analysis were not involved in the treatment of participants with low back pain.

In this study, the majority of the people in this study already had chronic low back pain at the onset of physiotherapy. The heterogeneity in the stages of low back pain may have influenced the results. For example, people with (sub)acute LBP with a complaint duration of less than twelve weeks generally tend to recover more quickly compared to people with chronic LBP [55]. To correct for these differences in back pain duration, the back pain duration was analysed as a covariate in the final model where low back pain duration showed a statistically significant association (*p* = 0.02) with the recovery slope of low back pain in model three in this study. This statistically significant association was not present at baseline, quadratic slope, or at the 12 months follow-up. The participating physiotherapy practices in this study were all specialized in spinal disorders. This is a possible explanation for the long low back pain duration at the onset of physiotherapy and the relatively high prevalence of radicular radiating leg pain [4, 28, 56, 57]. However, the radicular radiating low back pain group as well as the high risk SBST group were relatively small in our study population, so the radicular radiating group was merged with the non-radicular referred pain group and high risk SBST group was merged was merged medium risk group. This could have led to bigger mean differences between groups and may influenced the statistical results. Participants in this study received usual physiotherapy care according to the Dutch national guidelines of physiotherapy for low back pain [4]. The preference of physiotherapists and patients might have led to differences in the content of the physiotherapy treatments, which could have influenced the treatment fidelity. In the SBST is one question about complaints in the leg that might have caused some collinearity with the type of leg pain in the analysis. Another limitation is the small sample size (*n* = 22) of the participants with radicular radiating leg pain within the type of leg pain. Therefore, no powerful statements can be made about people with radicular radiating LBP. However, this sample does show which type of leg pain is most common in primary care, which appears to be low back pain without radicular appearance.

### Implications for practice and future research

The SBST was associated with the disability trajectory of low back pain. An implication for practice is that the outcome of the SBST provides a better understanding of the disability trajectories within the heterogeneous low back pain population in primary care. Patients with low back pain might benefit from tailored treatment based on the SBST outcome. For example, addressing modifiable psychosocial factors immediately might prevent people from non-recovery.

Future research might focus on researching the distinction between non-radiating low back pain, non-radicular referred leg pain, and radicular radiating leg pain in a larger cohort study.

## Conclusion

A medium or high risk-score for long-term disability using the STarT Back Screening Tool is associated with higher ODI disability scores at baseline, a different recovery slope in the recovery trajectory, and an impeded recovery at 12 months follow-up compared to a low risk-score in patients with low back pain. The type of leg pain was associated with the baseline ODI disability scores, while the type of leg pain was not associated with the slope in the disability trajectory or the ODI scores at 12 months follow-up. With the type of leg pain well-defined, the focus in practice should be less on categorising the type of leg pain. Instead, the focus should be shifted towards addressing psychosocial factors. The STarT Back Screening Tool is a useful tool to predict the disability trajectory in a heterogeneous group of people with low back pain in primary care and might, therefore, be recommended in future clinical practice guidelines.

### Electronic supplementary material

Below is the link to the electronic supplementary material.

Supplementary Material 1

## Data Availability

All data used and/or analysed during the current study are available from the corresponding author on reasonable request and with a signed data transfer agreement.
