# Supplementary material for: The association of the STarT Back Screening Tool and type of leg pain with low back pain disability trajectories: a prospective cohort study
Source: BMC Musculoskelet Disord. 2024 Mar 4;25:193. doi: 10.1186/s12891-024-07301-8 (PMC10910701; doi:10.1186/s12891-024-07301-8)
Supplement: Supplementary file 1 — Supplementary Material 1 [file 12891_2024_7301_MOESM1_ESM.docx]

Supplement A


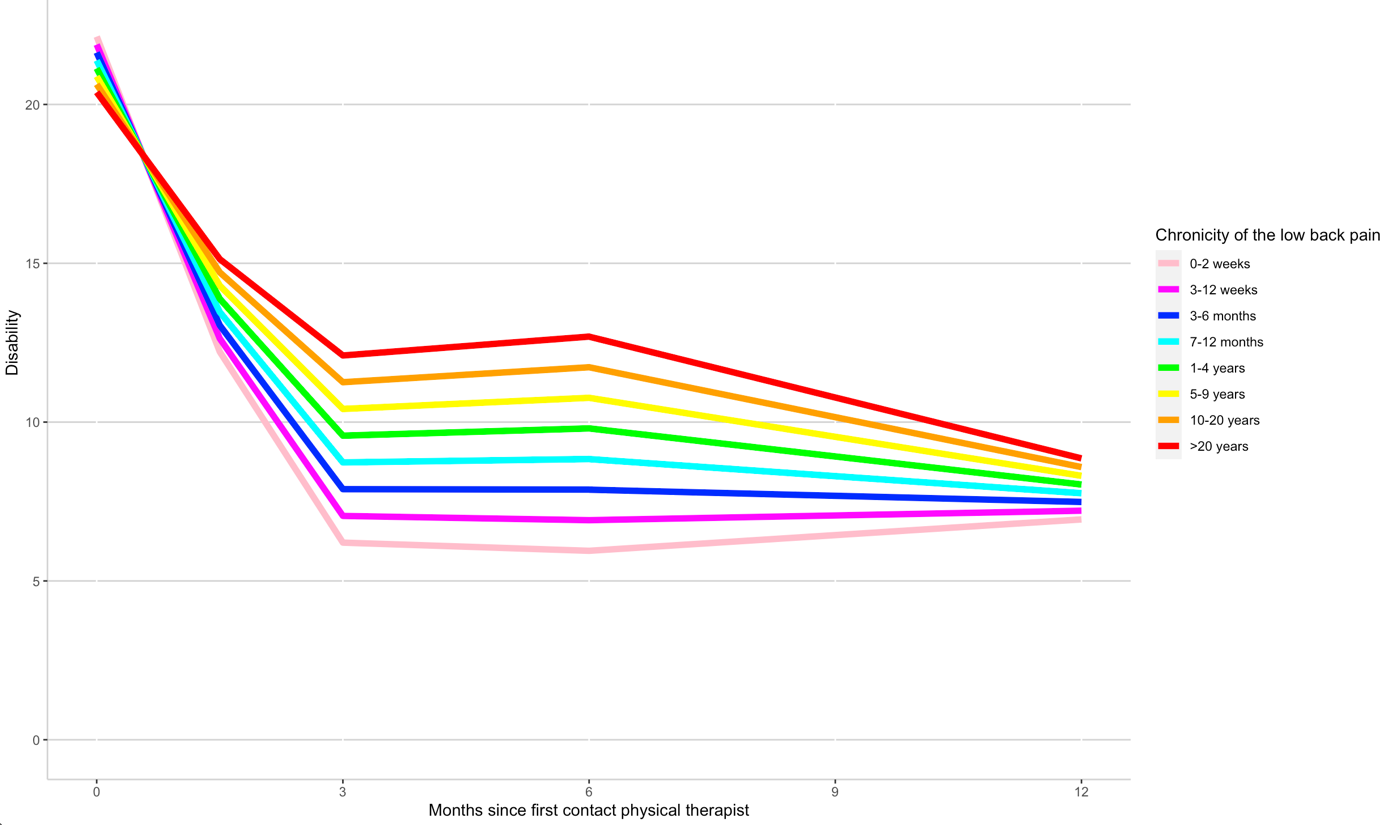


**Supplementary figure 6. Disability trajectory of back pain duration categories (model 3).** Disability measured with the Oswestry Disability Index (higher score indicates worse functioning).

| Supplementary table 6. Complete overview of all parameter estimates of the Results on baseline and slope for Fitting Different Individual Growth Models in Disability Trajectory Outcome Oswestry Disability Index (identical to table 2, model 3, but now also reporting the parameter estimates for all covariates) | | |  |  |  | | | |
| --- | --- | --- | --- | --- | --- | --- | --- | --- |
|  | | |  |  |  | | | |
|  | | | Model 3: Effects of Type of | | | |  |  |
|  | | | leg pain and SBST outcome | | | |  |  |
|  | | | (n=347) |  | | | |  |
| **Characteristic** | | | **Beta** | **95% CI^c^** | **p-value** | | | |
| ODI at BL | | | 12 | 9.4, 15 | <0.001 | | | |
| SBST category | | |  |  |  | | | |
| Low | | | — | — |  | | | |
| Medium^d^ | | | 11 | 7.8, 14 | <0.001 | | | |
| High | | | 22 | 17, 27 | <0.001 | | | |
| Type leg pain | | |  |  |  | | | |
| LBP | | | — | — |  | | | |
| Referred^e^ | | | 2.5 | -0.64, 5.7 | 0.12 | | | |
| Radicular | | | 15 | 9.0, 22 | <0.001 | | | |
| Baseline pain | | | 0.2 | -0.1, 0.5 | 0.42 | | | |
| Back pain duration | | | -0.3 | -0.7, 0.1 | 0.53 | | | |
| Gender | | | 0.9 | -0.6, 2.4 | 0.55 | | | |
| Age | | | 0.0 | -0.1, 0.0 | 0.56 | | | |
| Number of previous episodes of low back pain | | | 0.0 | -0.1, 0.0 | 0.73 | | | |
| Education level | | | 0.2 | -0.2, 0.6 | 0.73 | | | |
| linear change during first 6 months | | | -2.2 | -3.7, -0.67 | 0.005 | | | |
| SBST category * linear change during first 6 months | | |  |  |  | | | |
| Medium * linear change during first 6 months | | | -6.2 | -8.0, -4.4 | <0.001 | | | |
| High * linear change during first 6 months | | | -9.2 | -12, -6.3 | <0.001 | | | |
| Type leg pain * linear change during first 6 months | | |  |  |  | | | |
| Referred * linear change during first 6 months | | | -0.34 | -2.1, 1.4 | 0.7 | | | |
| Radicular * linear change during first 6 months | | | -1.3 | -4.9, 2.3 | 0.5 | | | |
| Baseline pain * linear change during first 6 months | | | 1.0 | 0.8, 1.2 | <0.001 | | | |
| Back pain duration * linear change during first 6 months | | | 0.5 | 0.3, 0.7 | 0.02 | | | |
| Gender * linear change during first 6 months | | | -1.1 | -2.0, -0.2 | 0.20 | | | |
| Age * linear change during first 6 months | | | 0.0 | 0.0, 0.0 | 0.30 | | | |
| Number of previous episodes of low back pain * linear change during first 6 months | | | 0.0 | 0.0, 0.0 | 0.68 | | | |
| Education level * linear change during first 6 months | | | -0.6 | -0.8, -0.4 | 0.02 | | | |
| quadratic change during first 6 months | | | 0.23 | 0.00, 0.45 | 0.053 | | | |
| SBST category * quadratic change during first 6 months | | |  |  |  | | | |
| Medium * quadratic change during first 6 months | | | 0.80 | 0.52, 1.1 | <0.001 | | | |
| High * quadratic change during first 6 months | | | 1.1 | 0.62, 1.5 | <0.001 | | | |
| Type leg pain * quadratic change during first 6 months | | |  |  |  | | | |
| Referred * quadratic change during first 6 months | | | 0.01 | -0.26, 0.28 | >0.9 | | | |
| Radicular * quadratic change during first 6 months | | | -0.04 | -0.59, 0.51 | 0.9 | | | |
| Baseline pain * quadratic change during first 6 months | | | -0.14 | -0.16, -0.12 | <0.001 | | | |
| Back pain duration * quadratic change during first 6 months | | | -0.05 | -0.08, -0.02 | 0.11 | | | |
| Gender * quadratic change during first 6 months | | | 0.20 | 0.1, 0.3 | 0.12 | | | |
| Age * quadratic change during first 6 months | | | 0.00 | 0.0, 0.0 | 0.27 | | | |
| Number of previous episodes of LBP * quadratic change during first 6 months | | | 0.00 | 0.0, 0.0 | 0.74 | | | |
| Education level * quadratic change during first 6 months | | | 0.09 | 0.06, 0.12 | 0.01 | | | |
| difference between ODI at 6 and 12 months | | | -2.5 | -5.1, 0.08 | 0.058 | | | |
| SBST category * difference between ODI at 6 and 12 months | | |  |  |  | | | |
| Medium * difference between ODI at 6 and 12 months | | | 1.1 | -2.1, 4.3 | 0.5 | | | |
| High * difference between ODI at 6 and 12 months | | | 5.7 | 0.64, 11 | 0.027 | | | |
| Type of leg pain * difference between ODI at 6 and 12 months | | |  |  |  | | | |
| Referred * difference between ODI at 6 and 12 months | | | 0.03 | -3.0, 3.1 | >0.9 | | | |
| Radicular * difference between ODI at 6 and 12 months | | | -1.9 | -8.6, 4.7 | 0.6 | | | |
| Baseline pain * difference between ODI at 6 and 12 months | | | -0.26 | -0.54, 0.02 | 0.36 | | | |
| Back pain duration * difference between ODI at 6 and 12 months | | | -0.69 | -1.07, -0.31 | 0.07 | | | |
| Gender * difference between ODI at 6 and 12 months | | | 0.33 | -1.15, 1.81 | 0.82 | | | |
| Age * difference between ODI at 6 and 12 months | | | 0.02 | -0.03, 0.07 | 0.74 | | | |
| Number of previous episodes of LBP * difference between ODI at 6 and 12 months | | | -0.01 | -0.07, 0.05 | 0.80 | | | |
| Education level * difference between ODI at 6 and 12 months | | | -0.23 | -0.66, 0.20 | 0.59 | | | |
| random intercept (sd) | | | 9.4 |  |  | | | |
| correlation random effects | | | -0.67 |  |  | | | |
| random linear slope (sd) | | | 1.4 |  |  | | | |
| residuals (sd) | | | 8.4 |  |  | | | |
| AIC | | | 10,39 |  |  | | | |
| BIC | | | 10,64 |  |  | | | |
|  | | |  |  |  | | | |
|  |  |  | | | |  |  |  |
|  |  |  | | | |  |  |  |
|  |  |  | | | |  |  |  |
